# Supplementary material for: Human SCARB2 Acts as a Cellular Associator for Helping Coxsackieviruses A10 Infection
Source: Viruses. 2023 Apr 8;15(4):932. doi: 10.3390/v15040932 (PMC10144829; doi:10.3390/v15040932)
Supplement: Supplementary file 1 [file viruses-15-00932-s001.zip › viruses-2317102-supplementary.pdf]

**Table S1.** List of the sequence of siRNA specific to target genes.

| siRNA | ID of siRNA | Target Gene | Sequences (5'->3') of siRNAs |
|---|---|---|---|
| K1 | S38393 | Human KRE-MEN1 | CCUUAGGGAUUGUCAUCAAtt |
| K2 | S38394 | Human KRE-MEN1 | CCAACAAACUCACCAUACAtt |
| S1 | S2651 | Human SCARB2 | CAAUAUCUGCUGUUAGCAAtt |
| S2 | S2652 | Human SCARB2 | GGACUAAUGAUGGAGACUAtt |

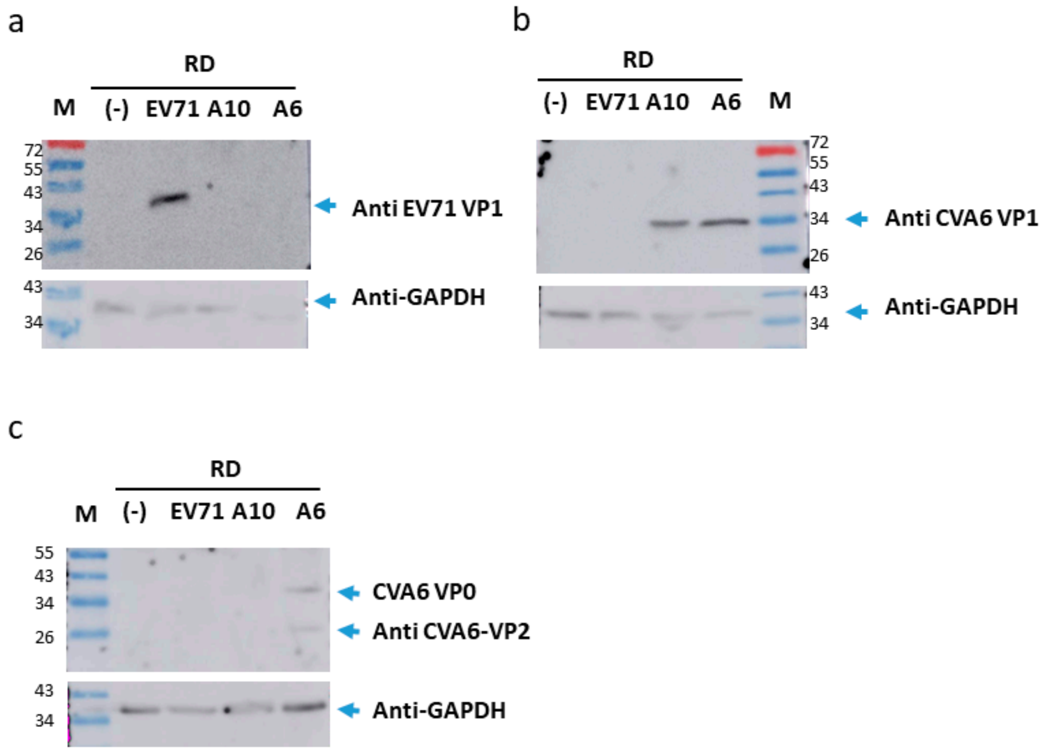


**Figure S1.** Cross-reactivity of polyclonal antibodies against CVA6 and CVA10. RD cells were individually infected with MOI=0.1 of EV71, CVA6, or CVA10 and then cultured for 24 h before lysate preparation. Lysate from the uninfected RD cells were also prepared as control. Lysates were subjected to Western blot using (**a**) mouse monoclonal antibody Mab979 against EV71, (**b**) polyclonal antibody against VP1 of CVA6 and CVA10, and (**c**) polyclonal antibody against VP0 and VP2 of CVA6 were shown. GAPDH as internal control of the each lysate was also detected by it’s specific antibody.

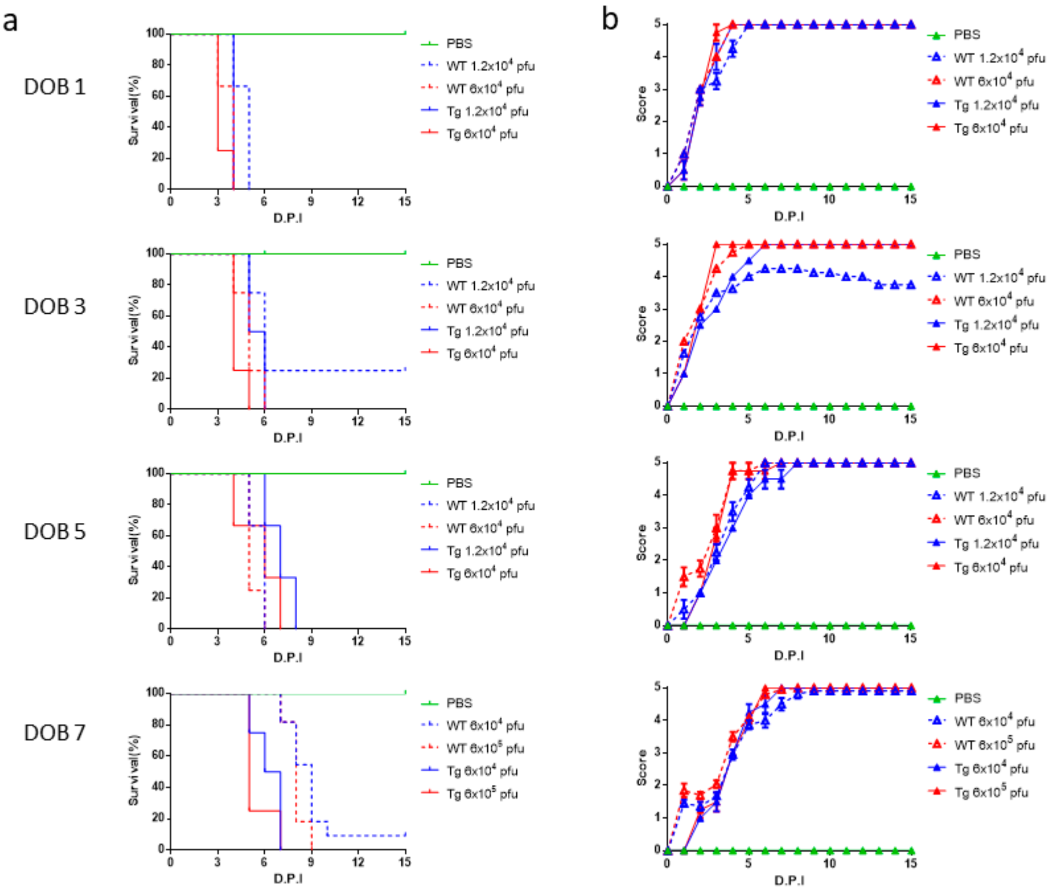


**Figure S2.** Pathogenesis and lethality of newborn hSCARB2-Tg mice infected with CVA10. Scoring of (**a**) disease score and (**b**) survival rate in 1, 3, 5, and 7 day of birth (DOB) of hSCARB2-Tg and non-Tg (WT) mice injected s.c. with different amounts of CVA10 and then monitored daily (day post infection, D.P.I.) for 15 days and assessed following the criteria described in the Materials and Methods section. The same age of Tg mice injected s.c. with PBS as control was included. Four mice per group were tested and bars corresponding to the mean disease score for each experimental group were shown.

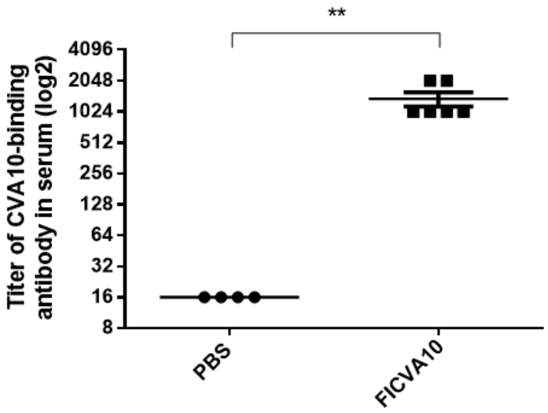


**Figure S3.** Induction of binding antibody against CVA10 by FI-CVA10.hSCARB2-Tg mice (6-8 weeks old) pre-immunized with PBS or two-dose of FI-CVA10 at a 2-week interval. Serum samples collected from individual Tg mice on day 28 post the second shot of FI-CVA10 were assayed for the titer of anti-CVA10 IgG by ELISA as described in the Materials and Methods section. The symbols ** was used to indicate $p < 0.01$.

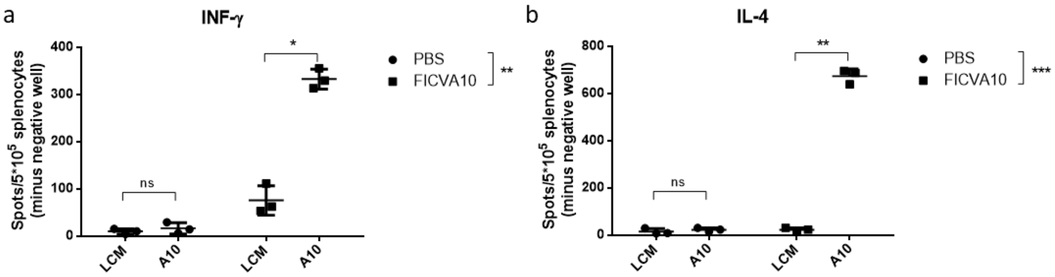


**Figure S4.** Induction of CVA10-specific splenocytic cytokines in FI-CVA10-immunized mice. hSCARB2-Tg mice (6–8 weeks old) were immunized twice s.c. with either PBS or FI-CVA10 at a 2-week interval and then sacrificed 14 days after the booster vaccine. Splenocytes were collected and cultured in the presence of cultured medium (LCM) or $10^7$ pfu/mL of UV-CVA10 (A10) for 24 h or 48 h and subjected to the (**a**) IFN-γ or (**b**) IL-4 ELISPOT assays, respectively, described in the Materials and Methods. Three mice per group were tested and bars corresponded to the mean of spots/5 × $10^5$ splenocytes for each experimental group were shown. The symbols *, **, ***, and ns were used to indicate $p < 0.05$, $p < 0.01$, $p < 0.005$, and no significant difference, respectively.
